# Activated Sludge Combined with Pervious Concrete Micro-Ecosystem for Runoff Rainwater Collection and Pollutant Purification

**DOI:** 10.3390/toxics12120838

**Published:** 2024-11-22

**Authors:** Yongsheng Zhang, Xuechen Jia, Pengfei Yuan, Bingqi Li, Wenyan Pan, Jianfei Liu, Weilong Zhao

**Affiliations:** 1 School of Civil Engineering, Henan Polytechnic University, Jiaozuo 454003, China; zhangyongsheng@hpu.edu.cn (Y.Z.); 18237790201@163.com (X.J.); yy8668@foxmail.com (P.Y.); a18272697752@163.com (B.L.); panwy@139.com (W.P.); jzitljf@foxmail.com (J.L.); 2 Henan Province Engineering Laboratory for Eco-Architecture and the Built Environment, Henan Polytechnic University, Jiaozuo 454000, China

**Keywords:** runoff rainwater, activated sludge, pervious concrete, ecosystem, microbial population

## Abstract

This study investigated the purification of pollutants in runoff rainwater by constructing a micro-ecosystem using waste-activated sludge (WAS) and riverbed sludge (RBS) as inoculums in combination with pervious concrete. The research results showed that the best hydraulic retention time (HRT) was 9 h. The COD and ammonia nitrogen (NH_4_^+^-N) removal of the waste-activated sludge ecosystem (WASE) was 62.67% and 71.21%, respectively, while the riverbed sludge ecosystem (RBSE) showed COD and NH_4_^+^-N removal percentages of 46.05% and 66.55%, respectively. The analysis of the genetic metabolism of microbial genes showed that the system was microbially enhanced with extensive and diverse populations. At the phylum level, the microorganisms responsible for degrading organic matter were mainly *Firmicutes* and *Actinobacteriota*. At the genus level, the *Trichococcus* genus was dominant in the WASE, while the *Dietzia, norank_f__Sporomusaceae* and *norank_f__norank_o__norank_c__BRH-c20a* genera were the central bacterial populations in the RBSE. The proliferation of phylum-level bacteria in the WASE was relatively large, and the genus-level bacteria demonstrated a better removal efficiency for pollutants. The overall removal effect of the WASE was better than that of the RBSE. The application analyses showed that a WASE is capable of effectively accepting and treating all rainfall below rainstorm levels and at near-full rainstorm levels under optimal removal efficiency conditions. This study innovatively used wastewater plant waste-activated sludge combined with pervious concrete to construct a micro-ecosystem to remove runoff rainwater pollutants. The system achieved pollutant removal comparable to that of pervious concrete modified with adsorbent materials. An effective method for the collection and pollutant treatment of urban runoff rainwater is provided.

## 1. Introduction

Due to rapid urbanization and industrialization, the initial runoff rainwater in cities is widely considered highly polluting [1,2]. Rainwater flows over urban surfaces, carrying large amounts of insoluble suspended solids, nitrogen, phosphorus, organic compounds, etc. [3,4]. Therefore, the burden on aquatic ecosystems is exacerbated, and there may also be potential risks to human health [5]. 

Bioretention basins are essential green facilities in sponge cities that are fully integrated into the urban environment and are widely used to address urban flooding and rainwater runoff pollution [6,7,8,9]. A bioretention basin reduces multiple pollutants in runoff rainwater, primarily through physical, chemical, and biological mechanisms [10,11]. Conventional bioretention basins use large quantities of sand and gravel, with high transportation and resource costs [12]. The cost issues can be addressed by adding fill to bioretention basins. One previously widely used type of fill was loess fill, but loess fill has collapsing properties [13]. Some scholars have attempted to modify loess as a filler using construction waste, concrete sand, wood chips mixed with loess, and sulfo-aluminate cement [14,15]. Not only does this improve the strength and stability of loess, but it also enhances the removal of pollutants from runoff rainwater. There is also literature on removing pollutants from rainwater by altering the substrate of artificial wetlands [16,17], which can be roughly divided into natural materials (marble, gravel, and sand), artificial materials (activated carbon and ceramic granules), and industrial by-products (slag and anthracite) [18,19,20,21,22,23,24]. In addition, there have been studies on removing various pollutants from rainwater by layering rare earth ceramic sand, ceramic grains, and oblique hair zeolite together as carrier materials applied to calamus artificial wetlands [25]. In summary, these methods require the preparation of appropriate fill and substrate to construct rainwater treatment facilities. Thus, researching a new purification method that is both simple and low-cost has become our primary concern.

In recent years, pervious concrete (PC) has been widely used in urban construction, as it allows runoff rainwater to pass through quickly [26,27]. Some researchers and scholars hope that pervious concrete can be used to mitigate the pollution of runoff rainwater. The ability of pervious concrete to remove pollutants is influenced by its pore structure and the adsorption capacity of the material [28,29]. Using this principle, researchers have added exogenous adsorption materials (steel slag, carbon nanotubes, zeolite powder, etc.) to eco-concrete. This approach relies on the adsorption capacity of the concrete itself to remove runoff rainwater pollutants [30,31,32]. Some scholars have constructed a brick masonry system with a frame structure to treat runoff rainwater using pervious concrete that incorporates gravel, zeolite, slag, volcanic rock, and iron filing material [33]. However, due to the price of highly adsorbent materials, the same problem of a high cost is faced. Microbial enhancement technology is being focused on as an ecological engineering tool [34,35,36]. The surface of pervious concrete can provide an environment for microorganisms to attach to and grow [37,38,39]. Therefore, removing pollutants during the passage of rainwater through the concrete can be achieved by increasing the number of functional microorganisms in the pervious concrete system. Moreover, this method can avoid the cost of purchasing and transporting fillers and does not require adsorbent materials to modify the concrete, which makes it valuable for research. 

In this study, an original proposal was made to use the micro-organisms in activated sludge to enhance pervious concrete. After reinforcement, it can be used as fill for rainwater pretreatment ponds, paving material for slope support, and wet grassing ditch paving material. Thus, this approach changes the previous use of pervious concrete as a floor covering material only. The concept of runoff rainwater collection coupled with pollutant removal represents a high level of innovation. A new research direction has been developed for the treatment of runoff rainwater. This study aimed to construct a system of pervious concrete reinforced by microorganisms combined in sludge. Research was conducted on the treatment efficacy of microbial-enhanced pervious concrete systems under different amounts of rainfall and different pollution levels of runoff rainwater in different regions. The principles of runoff rainwater pollutant removal from the system were explored in this study. The optimal pollutant removal efficiency and hydraulic retention time (HRT) for the operation of the system were derived. The biological enhancement mechanism of microorganisms in pervious concrete was revealed from the perspective of biological genetic metabolism. The evolutionary patterns of the microbial communities in biofilms were also assessed. The environmental benefits and practical application potential of microbe-enhanced pervious concrete systems were analyzed and summarized. Ultimately, these research results provide an effective way to purify and treat urban runoff rainwater.

## 2. Materials and Methods

### 2.1. Inoculum and Materials

The inoculums of the system were waste-activated sludge (WAS) and riverbed sludge (RBS), both of which were obtained from Jiaozuo, Henan, China. WAS was taken from the aerobic sludge of a sewage treatment plant using an A^2^/O (Anaerobic–Anoxic–Oxic) process, and RBS was taken from a riverbed 20 cm deep in the middle of a river. Pervious concrete was made using crushed stone in the 10–19 mm particle size range as aggregate. The cement was Polenta with a strength class of 42.5. A C1209 polycarboxylate superplasticizer was added to the material. The main materials of pervious concrete are shown in Table 1. The tap water came from Jiaozuo municipal water. The ecosystem construction and pervious concrete production used the same waste-activated sludge. The rainwater and culture solution used in the experiment were obtained synthetically. The drugs used for water distribution were glucose, ammonium chloride, sodium chloride, magnesium chloride, and disodium hydrogen phosphate in the compositional configuration shown in Table 2.

### 2.2. Production of Pervious Concrete

**Figure 1:**
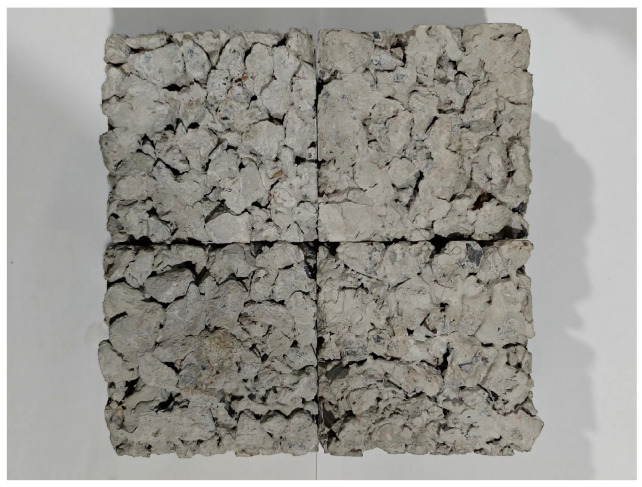
The finished product of pervious concrete after 28 d of curing.

The mass ratio of the WAS, cement, coarse aggregate and water in the pervious concrete was 1:11.5:41.7:3.4, and the proportion of water reducer added was 0.1%. The coarse aggregate was added to a mixer with 50% waste-activated sludge for 1 min. Then, cement, water reducer and the remaining 50% of the WAS were added and the concrete was mixed for 3 min. The freshly mixed concrete was poured into a 100 mm cube mold, compacted using vibration, and demolded after 1 d. Pervious concrete specimen blocks were made after being kept in the curing box at 30 °C for 28 d. The finished product is shown in Figure 1.

### 2.3. Micro-Ecosystem Construction

**Figure 2:**
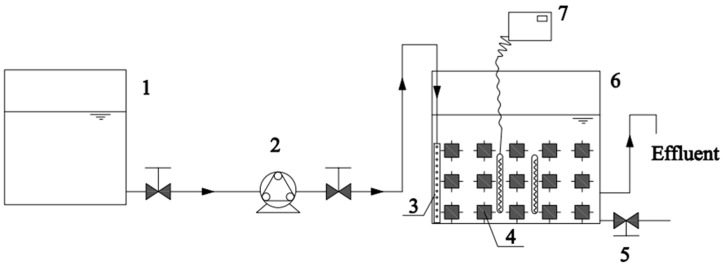
Schematic diagram of experimental rainwater purification device (1—water distribution tank; 2—peristaltic pump; 3—rainwater homogenizer; 4—cultivated pervious concrete specimen; 5—drain valve; 6—reaction tank; 7—heating rod and thermostatic controller).

A platform for activated sludge combined with pervious concrete was constructed to build the micro-ecosystem, as shown in Figure 2. To simulate the actual paving effect, the pervious concrete specimen blocks were placed closely to each other in a 55 cm × 55 cm × 50 cm container. The test blocks were arranged in 25 blocks per layer for 3 layers. The effective volume in the reactor was tested to be 9.2 L using the drainage method, because the test block occupied more volume. The water level at the container outlet was controlled at 17 cm. A peristaltic pump delivered the formulated synthetic rainwater to the rainwater homogenizer to achieve uniform water distribution in the reactor. The thermostat controller maintained the system’s operating temperature at 30 °C. The pH was not regulated during the system’s operation. Dissolved oxygen was obtained through the exchange between the water surface and the air, and there was no aeration device.

**Figure 3:**
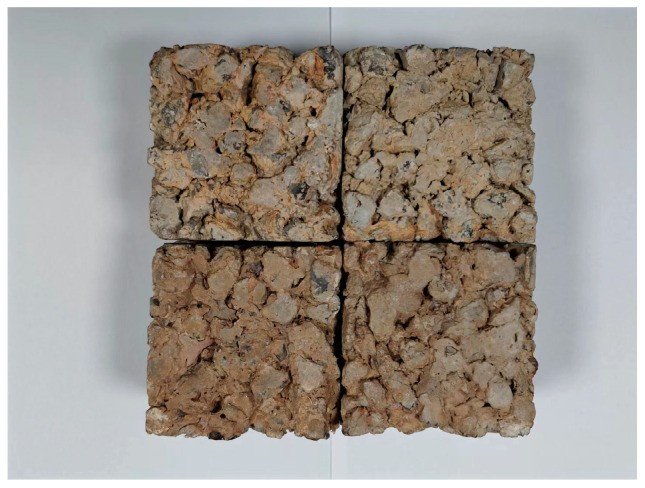
The finished pervious concrete after micro-ecosystem construction.

The culture solution was prepared according to the following parameters: COD, 500 mg/L; NH_4_^+^-N, 12.5 mg/L; TP, 2.5 mg/L; and pH, without artificial intervention. The reagents used in the culture solution were the same as those used in the synthesis of rainwater. Then, the WAS and RBS were added to the water body with a mass concentration of 15 g/L. The system with added waste-activated sludge is labeled as a waste-activated sludge pervious concrete micro-ecosystem (WASE), while the system with added riverbed sludge is labeled as a riverbed sludge micro-ecosystem (RBSE). The air pump continuously aerated to control the dissolved oxygen at 5–7 mg/L. After each day of collecting culture liquid, the drain valve was opened to let the liquid out. After emptying the liquid, it was necessary to add the same concentration of culture medium again to the water level line. The reactor needed to be continuously cultured for three weeks, until a biofilm appeared on the pervious concrete. After the effluent concentrations of COD and NH_4_^+^-N in the reactor stabilized, the cultivation was stopped. The finished pervious concrete after cultivation is shown in Figure 3.

### 2.4. Rainwater Purification Test

The rainwater purification experimental program included the influence of the HRT on the treatment effect and on the pollutant purification effect of the study. The specific rainwater purification experimental program is shown in Table 3.

(1) Effect of HRT on rainwater pollutant removal performance: The removal rates of COD and NH_4_^+^-N were tested at the HRTs of 6, 9, and 12 h, respectively. In this experiment, the concentrations of COD and NH_4_^+^-N in the synthetic rainwater were kept constant at 400 mg/L and 10 mg/L. All the other operating procedures were the same as for the pervious concrete cultivation process. Under each HRT condition, the reactor ran for a sufficient amount of time until steady-state conditions were achieved. The experiment used the RFE-1, RFE-2, and RFE-3 operating conditions in Table 3.

(2) Effect of influent concentration on rainwater pollutant removal performance: When the HRT was 9 h, the removal rate of pollutants was measured under the operating conditions of RFE-2, RFE-4, and RFE-5 in Table 3. At each influent concentration, the system was operated for a sufficient amount of time until it reached a stable state.

### 2.5. Test Methods

The COD and NH_4_^+^-N were measured according to the methods in the reported study [40,41]. The pH measurement was carried out using a pH meter modeled as PHSJ-4F from Shanghai INESA Scientific Instrument Co., Ltd., Shanghai, China. Each set of samples was tested three times to ensure the accuracy of the data.

The evolutionary analysis of microbial communities and gene functions was conducted using IlluminaMiSeq sequencing technology based on 16S rRNA genes. The evolution of microbial communities, such as archaea and bacteria, were analyzed. The bacterial primers, DNA extraction, and PCR amplification and sequencing were consistent with the methods used in the studies reported in [42,43,44]. The data were analyzed in the http://vip.majorbio.com/ URL (accessed on 17 December 2023) platform.

## 3. Results and Discussion

### 3.1. Effect of HRT on Rainwater Pollutant Removal Performance

**Figure 4:**
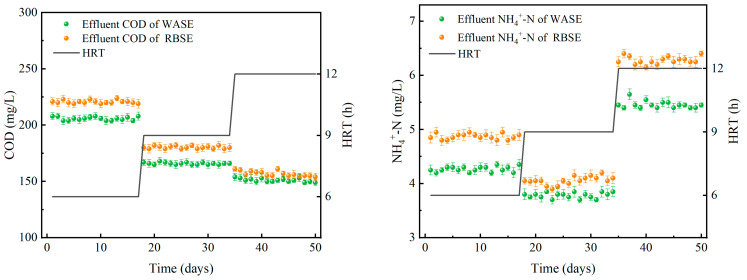
Effect of reactor on COD and NH_4_^+^-N nitrogen removal under different HRT conditions.

The hydraulic retention time (HRT) is the average time that wastewater remains in a treatment system. HRT directly affects the efficiency of microbial decomposition and the transformation of organic matter and pollutants in wastewater treatment processes. A suitable HRT helps maintain microbial community stability [45,46]. The treatment effects under different HRT conditions at an influent COD concentration of 400 mg/L and an NH_4_^+^-N concentration of 10 mg/L are shown in Figure 4 and Table 4. When the HRT was increased from 6 to 9 h, the COD removal in the WASE rose from 48.53% to 58.50%. When the HRT was further increased to 12 h, the COD removal increased to 62.22%. The COD removal in the RBSE system was similar to that in the WASE variation. As the HRT increased, the COD removal by both systems increased sequentially by 44.82%, 54.93%, and 60.78%, respectively. Due to the increase in the HRT, the biofilm’s contact time with the pollutant during operation was extended, thus increasing the pollutant removal rate [47]. When the HRT was increased to a particular stage, the improvement in the COD removal capacity was limited due to the limitation of microbial growth and metabolic activity.

When the HRT was increased from 6 h to 9 h, the NH_4_^+^-N removal in WASE rose from 57.32% to 62.18%. When the HRT was further increased to 12 h, the NH_4_^+^-N removal decreased to 45.44%. The NH_4_^+^-N removal in the RBSE system also showed a trend of increasing first and then decreasing. The maximum NH_4_^+^-N removal was 59.45% when the retention time was 9 h. At the same time, the pH in both systems was higher with increased NH_4_^+^-N removal. The highest pH value was 8.08 at an HRT of 9 h. The analysis suggested that, when the HRT was 6 h, organic matter could not be fully decomposed by the microorganisms due to the short contact time, resulting in a decrease in the removal rate [48]. When the HRT increased to 12 h, many microorganisms entered the death phase due to insufficient substrate. Microbial remains accumulated on the surface of the filling material, leading to an increase in anaerobic areas. The anaerobic reaction led to a decrease in the pH of the system and a decrease in the activity of nitrifying bacteria [49], which resulted in a reduction in NH_4_^+^-N removal [50]. 

An appropriate increase in the HRT can improve the removal of organic matter. However, when the HRT exceeds a certain value, the removal rate enhancement is limited due to the limitation of microbial growth and metabolic activities. The maximum COD and NH_4_^+^-N removal for both the WASE and RBSE systems was achieved at an HRT of 9 h. An HRT of 9 h was able to improve the pollutant removal efficiency while avoiding the negative effects of microbial metabolic activities.

### 3.2. Effect of Influent Concentration on Rainwater Pollutant Removal Performance

**Figure 5:**
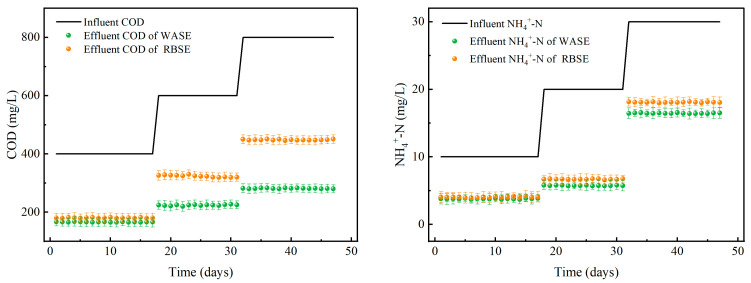
The effect of COD removal and NH_4_^+^-N removal by the reactor under different influent concentrations.

After verifying a suitable HRT of 9 h, the capacity of the sludge-constructed micro-ecosystem to treat runoff rainwater needed to be further determined. The treatment effects of the two systems under different concentrations of pollutants are shown in Figure 5. The COD removal in the WASE system gradually increased as the influent COD increased. When the influent COD concentrations were 400 mg/L, 600 mg/L, and 800 mg/L, the effluent COD concentrations were 58.50%, 62.67%, and 64.81%, respectively. In contrast, the COD removal in the RBSE gradually decreased with increasing influent COD. When the influent COD concentrations were 400 mg/L, 600 mg/L, and 800 mg/L, the effluent COD concentrations were 54.93%, 46.05%, and 43.95%, respectively. This phenomenon may have occurred since the activated sludge was screened and cultivated in wastewater treatment plants and had a robust adaptive capacity [51,52]. Within a specific range, an increase in the influent COD concentration led to an increase in COD removal in the reactor. RBS is a natural sludge with a wide variety and complex microbial species (described in detail in Section 3.3.1), and its adaptability is relatively weak. When the concentration increased, the microorganisms were poorly adapted, which caused a sharp decline in the removal rate [53]. 

When the influent NH_4_^+^-N concentrations were 10 mg/L and 20 mg/L, the removal rates of NH_4_^+^-N in the WASE were 62.18% and 71.21%, respectively, which were 2.73% and 4.66% higher than those in the RBSE. When the NH_4_^+^-N concentration was again increased to 30 mg/L, the NH_4_^+^-N removal in the WASE and RBSE decreased dramatically to 45.11% and 39.70%, respectively. The changes in the pH were the same as they were previously, all of which became larger as the NH_4_^+^-N removal increased. As the initial influent concentration increased, the microorganisms could fully utilize the contaminants, and the removal efficiency increased [47]. When the influent concentration increased again, the microbial load became too high. Due to the excessive consumption of oxygen dissolved by microorganisms, the pH decreased. A lower-pH environment could lead to a decrease in the activity of nitrifying bacteria, resulting in a decrease in the NH_4_^+^-N removal [54,55].

After comprehensively analyzing the results of the above test runs, the optimal HRT for reactor operation was determined to be 9 h. The best combined COD removal and NH_4_^+^-N removal was achieved at an HRT of 9 h, a COD concentration of 600 mg/L, and an NH_4_^+^-N concentration of 20 mg/L. Compared to the WASE system, the RBSE system performed better at all influent pollutant concentrations.

To fully assess the effectiveness of the pervious concrete ecosystem in rainwater pollutant removal, the results of this study were compared with those of other studies, as shown in Table 5. The primary materials selected for comparison were cement, red clay, and pervious concrete with other modified materials added. The comparison results showed that the pervious concrete ecosystem had better removal efficiency for pollutants. Compared to the more costly TiO_2_ pervious concrete, the effect on pollutants going out was comparable. Therefore, using pervious concrete to build the micro-ecosystem has certain advantages and a certain feasibility for pollutant removal from runoff rainwater.

### 3.3. Evolutionary Analysis of Microbial Community Structure

#### 3.3.1. Analysis of Microbial Community Structure

**Figure 6:**
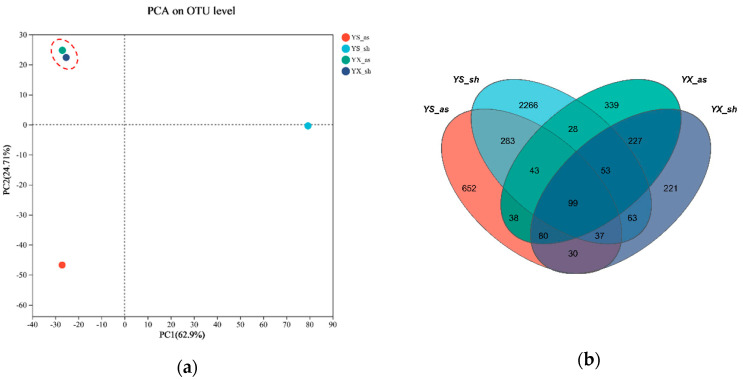
Principal component analysis of microbial community structure in the two systems before and after the operation (YS_as-initial WAS, YX_as-final WAS, YS_sh-initial RBS, and YX_sh-final RBS). (**a**) PCA analysis, (**b**) Venn.

The composition and abundance of microbial communities have a significant impact on treatment effectiveness. The sludge from different reactors with better experimental results was collected for testing, and the samples selected were the initial WAS (YS_as), the final WAS (YX_as), the initial RBS (YS_sh), and the final RBS (YX_sh). The microbial community structure analysis revealed the reasons for the purification effect of different species of mud after enhancement. The microbial community structure before and after the run was analyzed using principal component analysis, as shown in Figure 6.

Figure 6a represents the microbial community PCA analysis, and it can be seen that YS_as and YS_sh are far apart. This indicates a significant difference between the biological communities of WAS and RBS before and after the operation. It is worth noting that the biological communities of WAS and RBS were significantly different before the operation. However, after running and cultivating, the distance between YX_as and YX_sh was close. It is suggested that the samples had very similar biological communities [62]. Venn diagrams were used to visualize the number of unique species and shared species in the different microbial samples (Figure 6b) [63]. The OUT quantities of the YS_as, YS_sh, YX_as, and YX_sh samples were 1372, 2943, 962, and 878, respectively. The number of OUT shared by YS_as and YS_sth was 462, while the numbers for YX_as and YX_sh were 459. This phenomenon was consistent with the results presented by the PCA graph.

The results showed that there were relatively significant differences in the biological community structure due to the different sources of pre-operational sludge [64]. Under the same load conditions of pollutant types and concentrations, the microorganisms in different types of sludge exhibited different forms of environmental selection and different levels of adaptability to the environment. As a result, the biological communities of the WAS and RBS showed a high level of similarity after the operation [65]. 

#### 3.3.2. Evolutionary Analysis of Bacterial Communities

**Figure 7:**
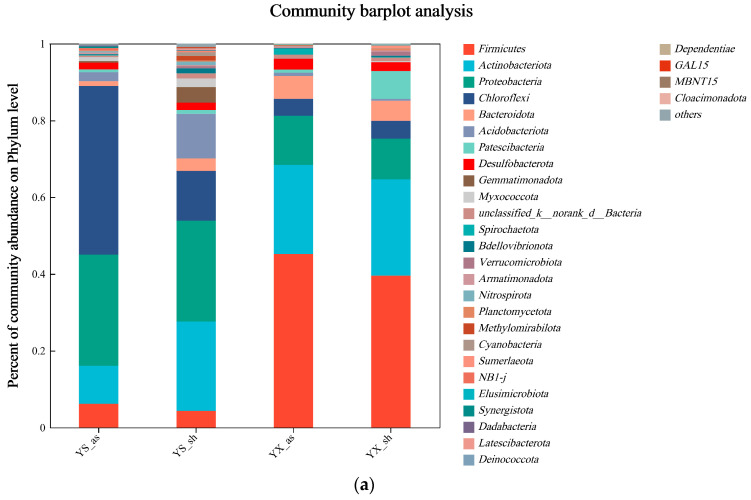

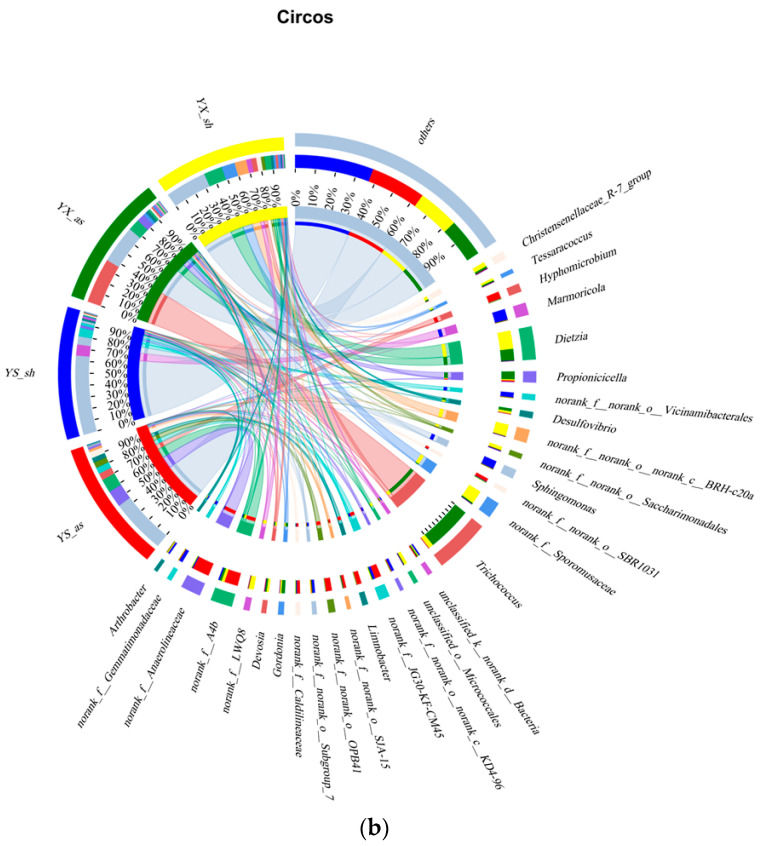
The analysis of the evolution of the microbial community structure in the two systems before and after the operation (YS_as-initial WAS, YX_as-final WAS, YS_sh-initial RBS, and YX_sh-final RBS). (**a**) Phylum-level bacterial communities, (**b**) Genus-level bacterial communities.

Figure 7 shows the results of the evolution of the microbial community structure in the different systems before and after the operation. In both systems, *Firmicutes*, *Actinobacterota*, *Proteobacteria*, and *Chloroflexi* were the main phyla, with abundances accounting for 4.34%~45.20%, 9.95%~25.07%, 10.61%~28.96%, and 4.67%~43.91%, respectively (Figure 7a). *Firmicutes* degraded organic matter into acetic acid, hydrogen gas, and carbon dioxide [66]. The *Firmicutes* content in the WAS and RBS before the operation was only 6.14% and 4.34%, respectively. The relative abundance of *Firmicutes* in the WAS and RBS at the end of the run increased by 39.06% and 35.2%, respectively. *Actinobacteriota* contributed to the decomposition of soluble organic matter. The abundance of *Actinobacteriota* in the samples after the run increased by 13.24% and 1.88% compared to before the run, respectively. *Proteobacteria* were responsible for the removal, denitrification, and degradation of COD from low-molecular-weight organic matter [67,68]. The abundance of *Proteobacteria* in the samples before and after the run decreased by 16.19% and 15.7%, respectively. The *Chloroflexi* was widely present as a flocculating skeleton within sludge colloidal flocs, contributing to sludge pelletization and the degradation of difficult-to-degrade organics [69,70]. The high abundance of *Chloroflexi* in YS_as, reaching 49.31%, also confirmed this. Because *Chloroflexi* is better suited to survive in an activated sludge environment, the enrichment abundance was reduced by 39.43% after a change in the operating environment. The analysis suggests that environmental conditions alter the abundance of functional bacteria, causing changes in the microbial community structure [71,72,73]. The presence of these communities makes microbially enhanced pervious concrete more effective at removing pollutants than conventional concrete. Moreover, as shown in Section 3.1, the activated sludge ecosystem had better pollutant removal rates than natural sludge, probably due to a greater increase in the relative abundance of *Firmicutes* and *Actinobacteriota* functional bacteria in the activated sludge [74].

The genus-level microbial communities in both systems were highly diverse, as shown in Figure 7b. For the activated sludge, *norank_f__Anaerolineaceae* and *norank_f__A4b* dominated before the operation, while *Trichococcu* dominated after the operation. After the operation, the relative abundances of *Trichococcus*, *Dietzia*, and *Propionicilla* in the activated sludge increased by 38.66%, 9.38%, and 5.49%, respectively, while the relative abundances of *norank_f__Anaerolineaceae* and *norank_f__A4b* decreased by 13.51% and 9.28%, respectively. Before the operation of riverbed sludge, *Marmoricola* and *Sphingomonas* were dominant. After the operation, *Dietzia*, *norank_f_ Sporomusaceae*, and *norank_f__norank_o__norank_c__BRH-c20a* were dominant, and *Marmoricola* and *Sphingomonas* almost wholly disappeared. Moreover, before the operation, the abundances of *Dietzia*, *norank_f__Sporomusaceae*, and *norank_f__norank_o__norank_c__BRH-c20a* in the riverbed sludge were extremely low, accounting for only 0.01%. However, after the operation, they proliferated until they became dominant. In contrast, the genus *Trichococcus*, which increased substantially in WAS, increased by only 4.14% in RBS. The results show that WAS exhibits significant differences from RBS at the genus level. The WAS mainly relied on the *Trichococcus* genus to remove pollutants [75], while the RBS mainly relied on *Dietzia*, *norank_f__Sporomusaceae*, and *norank_f__norank_o__norank_c__BRH-c20a* [76,77]. The previous results showed that activated-sludge-enhanced concrete was effective at removing pollutants. The reason was that, under suitable environmental conditions, *Trichococcus* had a better removal effect on pollutants than *Dietzia*, *norank_f__Sporomusaceae*, and *norank_f__norank_o__norank_c__BRH-c20a* [78,79]. 

Under the same pollutant species and concentration-loading conditions, the microorganisms in different types of sludge were selected by the environment and their own adaptations, which changed the abundance of functional bacteria and made the microbial community structure more similar. An increased abundance of *Trichococcus* populations with a higher pollutant treatment capacity resulted in better pollutant treatment in the corresponding systems.

### 3.4. Analysis of Activated Sludge Pervious Concrete Ecosystem Application and Comparison of Purification Effect with Other Studies

This study proposed the use of pervious concrete and activated sludge to construct micro-ecosystems. The micro-ecosystems showed a good treatment effect on pollutants in synthetic rainwater. From the previous studies, the best pollutant removal efficiency of the system was achieved for the following parameters: COD, 600 mg/L; NH_4_^+^-N, 20 mg/L; and HRT, 9 h. Primarily, after intensive microbial cultivation, the abundance and variety of functional microbial flora in the pervious concrete ecosystem increased, resulting in lower pollutant concentrations in rainwater. 

This study was also compared with the rainwater purification methods commonly used by other researchers to remove the same pollutants, as shown in Table 6. Compared to sewage-recycling artificial wetlands, bioretention ponds, and cistern-like artificial wetlands, the micro-ecosystem had a more substantial ability to remove pollutants. However, it was still weaker than the removal capacity of modified bioretention ponds and intensive artificial wetlands. The micro-ecosystem is a method with relatively balanced treatment effects. This study provided an efficient and highly applicable method for treating rainwater. At the same time, it provided a system operation strategy with a certain reference value.

Due to the complex changes in rainfall conditions in nature, an analysis of the potential application capabilities of the micro-ecosystem was conducted. After querying GB/T28592-2012 on the official China Meteorology website, the classification of the rainfall levels in different periods was determined, as shown in Table 7.

According to these research results, the WASE was more effective than the RBSE, with the best pollutant treatment achieved at an HRT of 9 h. At this time, the system flow rate was 24.5 L/d. Since the pervious concrete specimen arrangement was 0.5 m × 0.5 m, the coverage area was calculated to be 0.25 m^2^. Then, using the inlet flow rate, the coverage area, and a 1 d duration, the depth of water that the WASE system could accept per unit area in 24 h was calculated to be 98 mm. Regarding the classification of rainfall levels in Table 7, the WASE should be able to handle any amount of rainfall below the rainstorm level and close to all of the rainstorm levels under the best processing conditions. The pollutant content ranges for runoff rainwater here were referenced from the findings of this study. It can be seen from the summary calculation that the system proposed in this study had a strong level of acceptance and processing capacity, except for the relatively rare heavy rainstorm and extremely heavy rainstorm situations. The pervious concrete micro-ecosystem has good market application potential.

According to the summary of the previous study results, WASE showed more advantages compared to RBSE. For WASE, the microbial species in the activated sludge have been cultured and domesticated in the wastewater treatment plant. Although the overall microbial diversity was low, the abundance of functional microorganisms was high. Therefore, WASE has the advantages of fast startup, high pollutant degradation capacity and good adaptability to pollutant loads. Riverbed sludge was taken from natural ecosystems. For RBSE, microbial species were abundant, but functional microbial abundance was low, resulting in a low capacity to treat pollutants compared to WASE. However, the conclusions of this study were based on a laboratory environment with stable water intake and pollutant concentrations, without the influence of the external environment and other microbial populations. In practice, the natural environment often has many influencing factors. One of the most significant impacts is competition from exotic microbial populations, which can lead to a vulnerable position for functional microbes. The analysis suggests that RBSE will be more adaptable and competitive in the natural environment where it is actually applied. The comparison of the characteristics of WASE and RBSE is shown in Table 8.

Although this study verified that the use of activated sludge to strengthen pervious concrete is a viable method, research on the mechanism of pollutant treatment has not delved deep enough. It is hoped that this research will be extended by drawing on more research tools in the literature [87]. Furthermore, analyses of the microbial toxicity, respiration rate, nitrification rate, dehydrogenase activity, and other aspects of the mechanism should be carried out. It is hoped that more efficient, targeted, and cost-effective methods of treating runoff rainwater can be developed.

## 4. Conclusions

In this study, a pervious concrete micro-ecosystem was constructed. An experiment was conducted on the purification of rainwater by the activated sludge pervious concrete micro-ecosystem. The micro-ecosystem demonstrated specific improvements in removing pollutants from rainwater. The ecosystem fostered by sludge with a high microbial activity and specificity improved the removal efficiency of pollutants from rainwater. Before and after the operation, there were significant changes in the microbial community structure and functional bacterial genera in the micro-ecosystem. The richness of microbial communities with a high efficiency in degrading organic matter increased, improving the pollutant removal efficiency for rainwater. Ultimately, the activated sludge combined with the pervious concrete micro-ecosystem demonstrated a good pollutant removal efficiency for rainwater under most rainfall conditions.

## Figures and Tables

**Table 1:** The main materials of pervious concrete.

Materials	Coarse Aggregate	Cement	Water	Activated Sludge	Water Reducer
	g				
Mass	1500	414	121.5	36	0.45

**Table 2:** Configuration of synthetic rainwater constituent concentrations.

COD	NH_4_^+^-N	C_6_H_12_O_6_	NH_4_Cl	NaCl	MgCl_2_	K_2_HPO_4_
mg/L						
400	10	373.8	38.5	20.0	2.5	3.7
600	20	560.8	76.9	25.0	3.0	5.6
800	30	747.7	115.4	30.0	3.5	7.5

**Table 3:** The experimental program for rainwater purification.

Number	Influent COD (mg/L)	Influent NH_4_^+^-N (mg/L)	HRT (h)
RFE-1	400	10	6
RFE-2	400	10	9
RFE-3	400	10	12
RFE-4	600	20	9
RFE-5	800	30	9

**Table 4:** COD and NH_4_^+^-N removal by activated sludge and riverbed sludge reactors.

Number	Activated Sludge Reactor			Riverbed Sludge Reactor		
	COD Removal	NH_4_^+^-N Removal	Final pH	COD Removal	NH_4_^+^-N Removal	Final pH
REF-1	48.53%	57.32%	8.13 ± 0.23	44.82%	51.29%	7.99 ± 0.15
REF-2	58.50%	62.18%	8.28 ± 0.36	54.93%	59.45%	8.08 ± 0.27
REF-3	62.22%	45.44%	7.49 ± 0.18	60.78%	37.22%	7.37 ± 0.12
REF-4	62.67%	71.21%	8.76 ± 0.21	46.05%	66.55%	8.34 ± 0.25
REF-5	64.81%	45.11%	7.29 ± 0.16	43.95%	39.70%	7.45 ± 0.19

**Table 5:** Comparison of pollutant removal effects of pervious concrete micro-ecosystem with other studies.

Materials	COD Removal	NH_4_^+^-N Removal	Refs.
Recycled aggregates	52%	42%	[56]
Ceramics	10%	20%	[57]
Steel slag	14%	32%	[57]
Mineral adsorbents and biochar	31%	20%	[58,59]
Composite cement	35%	47%	[60]
General cement	22%	44%	[60]
Composite cement and red clay	31%	55%	[60]
TiO_2_	69%	78%	[61]
Pervious concrete micro-ecosystem	63%	71%	This study

**Table 6:** Comparison of the micro-ecosystem with other rainwater purification systems.

System Type	COD Removal	NH_4_^+^-N Removal	Ref.
Bioretention basin	65%	63%	[80]
Modified bioretention basin	76%	89%	[81]
Cistern-like artificial wetland	42%	51%	[82]
Stabilization ponds and artificial wetland	32%	77%	[83]
High-throughput artificial wetland	65%	20%	[84]
Intensive artificial wetland	84%	63%	[85]
Sewage-recycling artificial wetland	66%	55%	[86]
Pervious concrete micro-ecosystem	63%	71%	This study

**Table 7:** Classification of rainfall levels in different periods.

Rainfall Levels	Time Period Rainfall (mm)	
	12 h	24 h
Light rainfall (scattered drizzle)	<0.1	<0.1
Light rain	0.1~4.9	0.1~9.9
Moderate rain	5.0~14.9	10.0~24.9
Heavy rain	15.0~29.9	25.0~49.9
Rainstorm	30.0~69.9	50.0~99.9
Heavy rainstorm	70.0~139.9	100.0~249.9
Extremely heavy rainstorm	≥140.0	≥250.0

**Table 8:** Comparison of the characteristics of WASE and RBSE.

Characterization	WASE	RBSE
Microbial diversity	Low	High
Functional microorganisms abundance	High	Low
Rainwater treatment capacity	High	Low
Start-up speed	Fast	Slow
Pollutant load adaptability	Strong	Weak
Pollutant degradation capacity	Strong	Weak
Natural environment adaptation	Weak	Strong

## Data Availability

The data that support the findings of this study are available from the corresponding author upon reasonable request.
